# Peripheral blood markers predict prognosis and irAEs of stage IV driver gene-negative lung adenocarcinoma treated with ICIs

**DOI:** 10.3389/fimmu.2025.1538392

**Published:** 2025-03-28

**Authors:** Yu Li, Lei Cao, Lei Liu, Yawen Ding, Feng Cao

**Affiliations:** ^1^ Department of Radiation Oncology, The Fourth Hospital of Hebei Medical University, Shijiazhuang, China; ^2^ Department of Geriatric Respiratory, Hebei General Hospital, Shijiazhuang, China; ^3^ Department of Thoracic Surgery, The Fourth Hospital of Hebei Medical University, Shijiazhuang, China; ^4^ Clinical Laboratory, The Fourth Hospital of Hebei Medical University, Shijiazhuang, China

**Keywords:** lung adenocarcinoma, immunotherapy, prognosis, irAEs, PLR, LMR

## Abstract

**Objective:**

This study aims to evaluate the prognostic significance of peripheral blood biomarkers in relation to outcomes and immune-related adverse events (irAEs) among patients with stage IV driver gene-negative lung adenocarcinoma receiving treatment with immune checkpoint inhibitors (ICIs).

**Methods:**

We conducted a retrospective analysis of clinicopathological data from 102 patients diagnosed with stage IV driver gene-negative lung adenocarcinoma who were treated with ICIs at the Fourth Hospital of Hebei Medical University between January 1, 2019, and December 31, 2023. We employed the Kaplan-Meier method to perform a univariate analysis of progression-free survival (PFS) and overall survival (OS), generated survival curves, and assessed differences in survival between groups using the log-rank test. The Cox regression model was utilized for multivariate analysis of PFS and OS. Additionally, we assessed the predictive value of peripheral blood markers for irAEs using logistic regression models.

**Results:**

The 1-, 3-, and 5-year PFS rates for the cohort of 102 patients were recorded at 35.3%, 3.9%, and 0%, respectively; similarly, the OS rates at these time points were observed to be 66.7%, 8.8%, and 2.9%. Multivariate analysis identified that the prognostic nutritional index (PNI) and metastatic status served as independent prognostic factors influencing PFS outcomes in this patient population; furthermore, D-dimer levels, PNI, metastatic status, and the occurrence of irAEs emerged as independent prognostic indicators impacting OS rates among patients. Subsequent logistic regression analysis revealed that both platelet-to-lymphocyte ratio (PLR) and lymphocyte-to-monocyte ratio (LMR) functioned as independent predictors of irAEs with statistical significance (P=0.032; P=0.02).

**Conclusion:**

For patients with stage IV driver gene-negative lung adenocarcinoma undergoing ICI therapy, PNI and metastatic status can serve as initial predictors of PFS. Additionally, D-dimer levels, PNI, metastatic status, and the presence of irAEs can initially predict OS, aiding in the identification of populations that may benefit from ICI therapy in clinical practice. Furthermore, our findings indicate a need for heightened attention to PLR and LMR concerning the occurrence of irAEs.

## 1 Introduction

Lung cancer remains the foremost cause of cancer-related mortality globally (1). Among its subtypes, lung adenocarcinoma constitutes approximately 40% of lung cancer cases, with a steadily increasing incidence; most patients present with distant metastases at the time of diagnosis (2, 3). The advent and ongoing advancement of targeted therapies have significantly enhanced survival rates for patients with stage IV mutant lung adenocarcinoma over the past decade. Furthermore, the introduction of immunotherapy for stage IV lung adenocarcinomas characterized by driver-negative mutations has also conferred notable survival advantages (4–6), prompting heightened interest in evaluating immunotherapy efficacy, population benefits, and potential adverse effects. Identifying patient populations that may benefit from these treatments to optimize therapeutic outcomes and predicting adverse reactions for early intervention have emerged as pressing challenges requiring resolution. Currently, PD-L1 quantification via immunohistochemical assays stands as the most extensively validated and accepted biomarker (7), yet it is not without its limitations (8–10). The efficacy of immune checkpoint inhibitors (ICIs) is not directly proportional to the expression levels of biomarkers, and a significant challenge lies in the accessibility of specimens as well as the feasibility of dynamic monitoring. Recent studies have indicated that peripheral blood indicators can serve as predictive markers for the efficacy of ICI therapy, demonstrating prognostic value in stage IV non-small cell lung cancer (NSCLC) (11). However, there are currently no effective biomarkers available to predict treatment efficacy or the occurrence of irAEs in patients with driver-negative lung adenocarcinoma undergoing ICI therapy. Consequently, this study aims to elucidate further prognostic factors associated with ICIs in treating stage IV driver gene-negative lung adenocarcinoma and to explore peripheral blood markers for predicting irAEs. The ultimate goal is to provide clinicians with more precise prognosis assessments and individualized treatment strategies, thereby enhancing therapeutic outcomes and improving patients’ quality of life.

## 2 Materials and methods

### 2.1 Patients’ selection and general information

Lung cancer patients diagnosed and treated in the Fourth Hospital of Hebei Medical University from January 1, 2019, to December 31, 2023, were collected. Inclusion criteria: (1) adenocarcinoma at initial diagnosis at the Fourth Hospital of Hebei Medical University by histopathology; (2) complete systemic examination data and complete clinicopathologic data; (3) according to the 2015 International Association for the Study of Lung Cancer (IASLC) newly revised of the 8th edition of the TNM staging criteria for lung cancer (12) for patients with stage IV; (4) receiving at least twice consecutive ICIs.

Exclusion criteria: (1) history of other malignant tumors; (2) presence of driver gene mutations; (3) antitumor treatment history; (4) patients who had received only 1 cycle of ICIs; and (5) patients with incomplete clinical data.

### 2.2 Observation indexes

Sex, age, smoking history, drinking history, distant metastatic sites, number of metastases, tumor markers, hematological indexes, treatment modalities, PFS and OS were collected from stage IV lung adenocarcinoma patients who were negative for driver genes and treated with ICIs.

PFS was defined as the time interval from the start of treatment to the onset of tumor progression (in any respect) or death (from any cause).

OS was defined as the time interval from the start of treatment to death (for any reason) or the last recorded follow-up.

In this study, we used the European consensus on synchronous oligometastasis in lung adenocarcinoma as a criterion for defining oligometastasis, i.e., the number of metastatic tumor sets was less than five and the number of metastatic organs was not more than three (13).

NLR (neutrophil to lymphocyte ratio) was defined as the ratio of peripheral blood neutrophil count to lymphocyte count.

PLR is defined as the ratio of platelet count to lymphocyte count in peripheral blood.

LMR was defined as the ratio of lymphocyte count to monocyte count in peripheral blood.

PNI was calculated as serum albumin (g/L) + 5 × total peripheral blood lymphocyte count (×10^9^/L).

### 2.3 Follow-up

Patients were monitored through telephone consultations and outpatient evaluations. The follow-up period ended on July 31, 2024, with a median follow-up period of 16.5 months. At the conclusion of the follow-up period, there were 55 recorded deaths, 38 survivors, and 9 cases lost to follow-up, resulting in a follow-up rate of 91.2%.

### 2.4 Statistical methods

Statistical analyses were conducted using SPSS version 27.0. We employed the Kaplan-Meier method to perform a univariate analysis of PFS and OS, generated survival curves, and assessed differences in survival between groups using the log-rank test. The Cox regression model was utilized for multivariate analysis of PFS and OS. Univariate and multivariate analyses regarding factors influencing irAEs were performed through logistic regression models, with a significance threshold set at P < 0.05.

## 3 Results

### 3.1 General clinical data of stage IV driver gene-negative lung adenocarcinoma patients treated with ICIs

From January 1, 2019, to December 31, 2023, 102 cases of stage IV driver gene-negative lung adenocarcinoma patients treated with ICIs were admitted to our hospital. Among them, 76 cases were male and 26 cases were female; age ranged from 35 to 78 years old, with a median age of 65 years old; there were 70 cases of oligometastasis and 32 cases of extensive metastasis; there were 77 cases of systemic therapy only (including immunotherapy and chemotherapy) and 25 cases of systemic therapy combined with radiotherapy; there were 37 cases of immunotherapy adverse events and 65 cases of no immunotherapy adverse events (**Table 1**).

**Table 1:** Characteristics of 102 patients with stage IV driver gene-negative lung adenocarcinoma treated with ICIs.

Feature	N (%)	
Sex		
Male	76	74.5%
Female	26	25.5%
Age		
≤ 65	55	53.9%
> 65	47	46.1%
Smoking		
No	39	38.2%
Yes	63	61.8%
Drinking		
No	63	61.8%
Yes	39	38.2%
Metastatic status		
Oligometastasis	70	68.6%
Extensive metastasis	32	31.4%
Radiotherapy		
No	77	75.5%
Yes	25	24.5%
irAEs		
No	65	63.7%
Yes	37	36.3%

### 3.2 Serum tumor markers

The normal reference value of carcinoembryonic antigen (CEA) was 0-5ng/ml, the normal reference value of soluble cytokeratin (CYFRA21-1) was 0-3.3ng/ml, the normal reference value of neuron-specific enolase (NSE) was 0-16.3ng/ml, and the normal reference value of squamous epithelial cell carcinoma antigen (SCC) was 0-1ng/ml. according to the patients’ pre-treatment values of the four tumor markers were divided into normal and elevated groups, respectively (**Table 2**).

**Table 2:** Baseline Peripheral blood markers of 102 patients with stage IV driver gene-negative lung adenocarcinoma treated with ICIs.

Variable	N (%)	
CEA		
≤ 5	39	38.2%
> 5	63	61.8%
CYFBA21-1		
≤ 3.3	29	28.4%
>3.3	73	71.6%
NSE		
≤ 16.3	56	54.9%
> 16.3	46	45.1%
SCC		
≤ 1	75	73.5%
> 1	27	26.5%
D-dimer		
≤ 0.5	67	65.7%
> 0.5	35	34.3%
NLR		
≤ 5	79	77.5%
> 5	23	22.5%
PLR		
≤ 185	66	64.7%
> 185	36	35.3%
LMR		
≤ 4	53	52.0%
> 4	49	48.0%
PNI		
≤ 45	29	28.4%
> 45	73	71.6%

### 3.3 Hematological indicators

The normal reference value of D-dimer was 0-0.5 mg/L. According to the published literature, we defined the cut-off values of NLR, PLR, LMR, and PNI as 5, 185, 4, and 45, respectively (14–17). The patients were divided into two groups based on their respective cut-off values, respectively (**Table 2**).

### 3.4 Univariate and multivariate analysis of PFS

**Figure 1:**
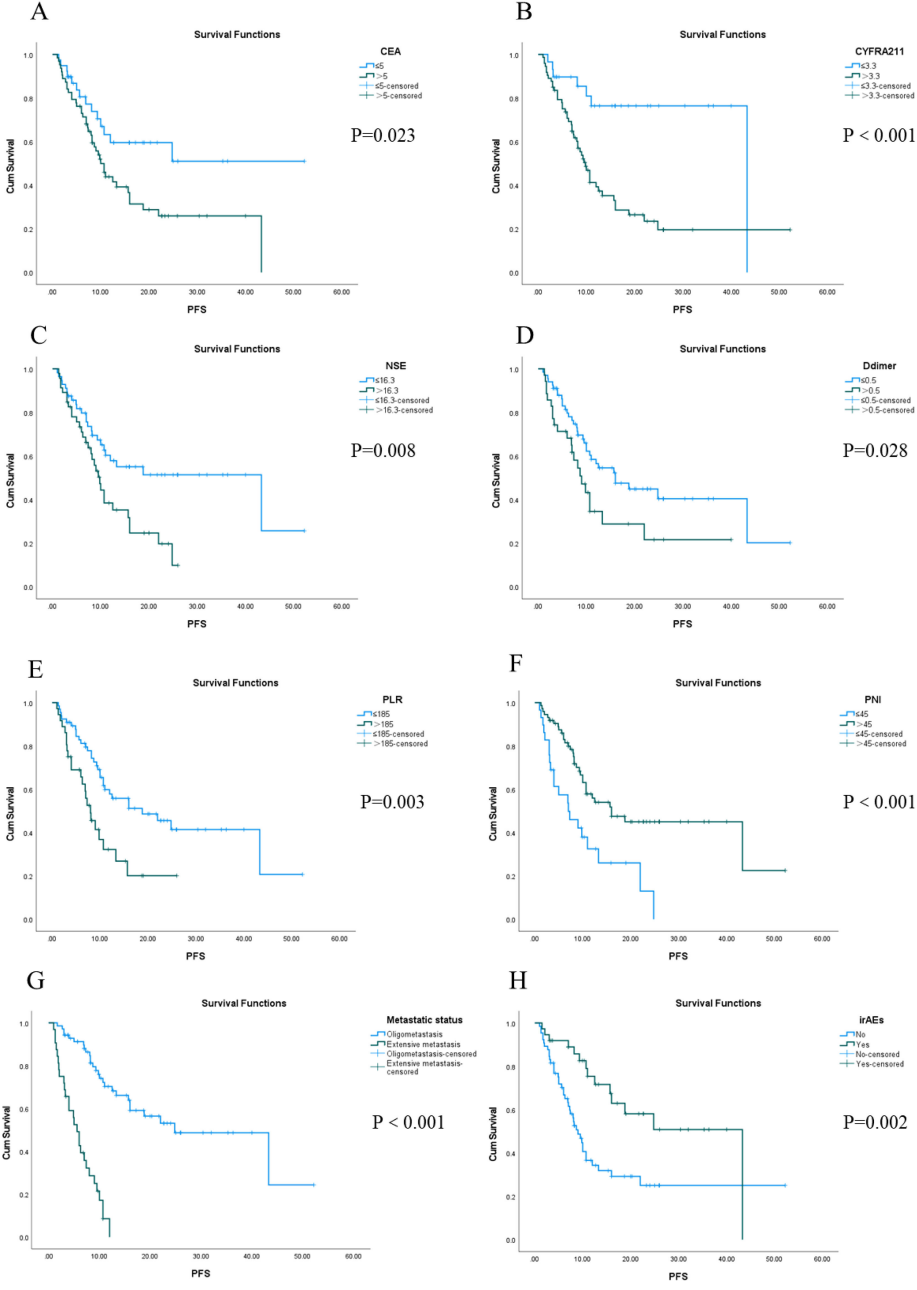
Kaplan-Meier survival analysis of PFS in 102 patients with stage IV driver gene-negative lung adenocarcinoma treated with ICIs from **(A–H)**.

The longest PFS observed in our cohort was 52.2 months, while the shortest was 1.0 month, resulting in a median PFS of 13.2 months. The 1-, 3- and 5-year PFS rates in the whole group were 35.3%, 3.9% and 0%, respectively. Univariate analysis identified several factors influencing PFS in this patient population, including CEA levels, CYFRA21-1, NSE, D-dimer levels, PLR, PNI scores, metastatic status, and the presence of irAEs (see **Figure 1**). Furthermore, Cox regression analysis revealed that both PNI and metastatic status were independent predictors of PFS for these patients. Notably, among individuals with stage IV driver-negative lung adenocarcinoma receiving immune checkpoint inhibitors (ICIs), those with a PNI score >45 experienced significantly longer PFS compared to those with a score ≤ 45 (P = 0.027). Additionally, patients presenting with oligometastasis demonstrated significantly prolonged PFS relative to those with extensive metastasis (P < 0.001) (**Table 3**).

**Table 3:** Cox regression analysis of influencing factors of PFS in 102 patients with stage IV driver gene-negative lung adenocarcinoma treated with ICIs.

Variable	HR	95.0%CI lower limit	95.0%CI upper limit	P value
CEA	1.717	0.883	3.340	0.111
CYFRA21-1	2.074	0.879	4.897	0.096
NSE	1.080	0.596	1.958	0.799
D-dimer	0.662	0.338	1.295	0.229
PLR	1.212	0.596	2.465	0.595
PNI	0.438	0.211	0.909	0.027
Metastatic status	5.765	2.844	11.685	<0.001
irAEs	0.663	0.323	1.363	0.264

### 3.5 Univariate and multivariate analysis of OS

**Figure 2:**
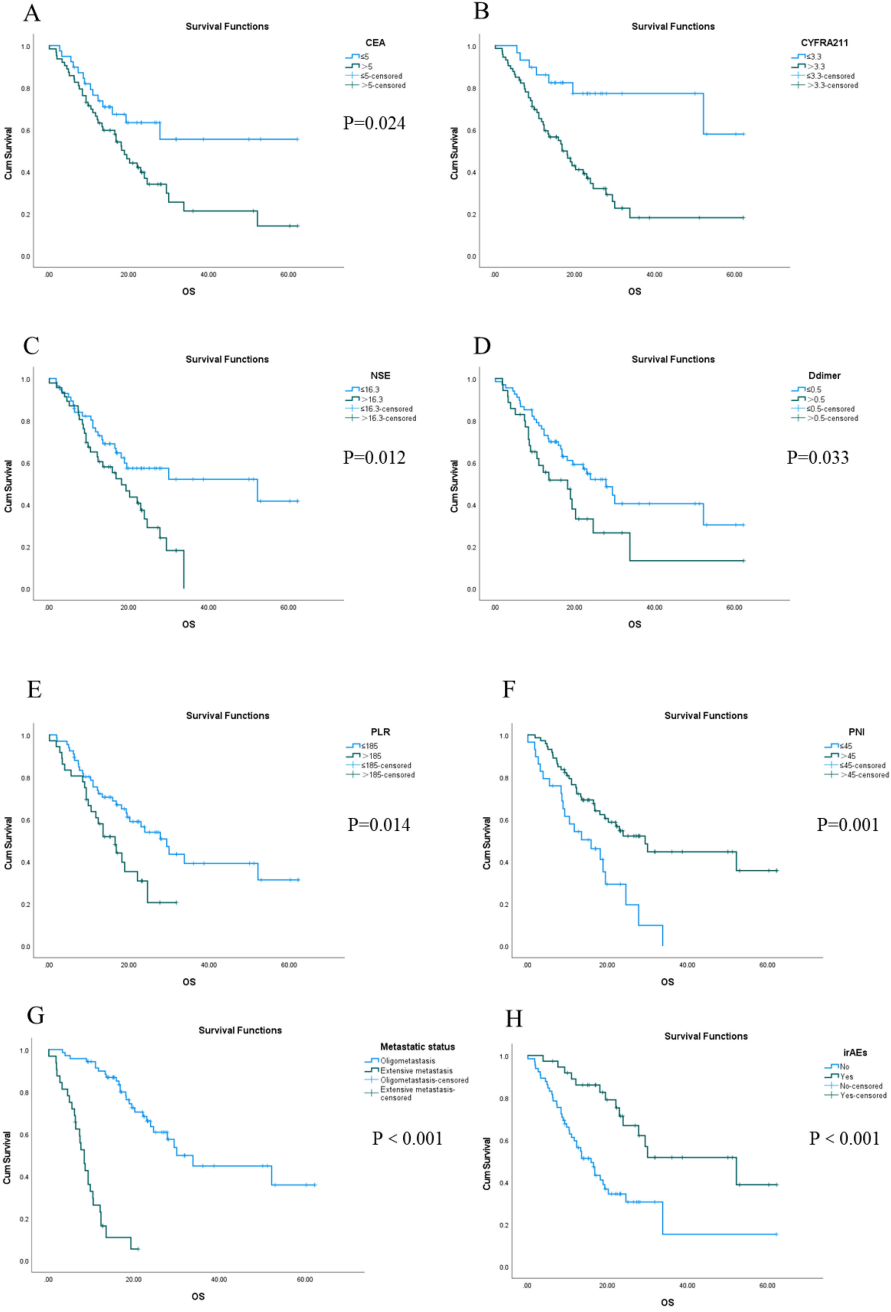
Kaplan-Meier survival analysis of OS in 102 patients with stage IV driver gene-negative lung adenocarcinoma treated with ICIs from **(A–H)**.

The longest OS recorded was 62.3 months, while the shortest OS was 1.5 months, and the median OS stood at 16.5 months. The 1-, 3-, and 5-year OS rates were 66.7%, 8.8%, and 2.9%, respectively. Univariate analysis revealed that factors such as CEA, CYFRA21-1, NSE, D-dimer, PLR, PNI, metastatic status, and the presence or absence of irAEs were statistically significant (**Figure 2**). Furthermore, multivariate Cox regression analysis identified D-dimer levels, PNI scores, metastatic status, and the occurrence of irAEs as independent prognostic factors influencing OS in this patient cohort. Notably, the OS of patients with normal D-dimer, PNI> 45, oligometastasis, and irAEs was longer than that of patients with high D-dimer, PNI ≤ 45, extensive metastasis, and no irAEs. (all P < 0.05) (**Table 4**).

**Table 4:** Multivariate Cox regression analysis of OS influencing factors in 102 patients with stage IV driver gene-negative lung adenocarcinoma treated with ICIs.

Variable	HR	95.0%CI lower limit	95.0% CI upper limit	P value
CEA	1.829	0.931	3.591	0.080
CYFRA21-1	2.180	0.920	5.165	0.077
NSE	1.151	0.632	2.098	0.645
D-dimer	0.390	0.182	0.837	0.016
PLR	0.765	0.367	1.597	0.476
PNI	0.342	0.162	0.724	0.005
Metastatic status	10.438	4.834	22.542	<0.001
irAEs	0.432	0.207	0.901	0.025

### 3.6 Analysis of the occurrence of irAEs and influencing factors

A total of 37 patients experienced irAEs, while 65 patients did not. Among the 37 patients with irAEs, there were 36 cases classified as Grade 1 to Grade 2, primarily consisting of immune-associated rashes (14 cases), hypothyroidism (11 cases), reactive cutaneous capillary hyperplasia (5 cases), nephrotoxicity (4 cases), and immune-associated pneumonia (2 cases). There was one case of Grade 3 irAE, which was also an instance of immune-associated pneumonia. The presence or absence of irAEs was analyzed using a one-way logistic regression for various biomarkers including CEA, CYFBA21-1, NSE, SCC, D-dimer, NLR, PLR, LMR, and PNI. The results demonstrated that NLR, PLR, and LMR were statistically significant variables, as presented in **Table 5**; those with p-values less than 0.05 were included in the multivariate analysis. Subsequent multivariate logistic regression analyses indicated that both PLR and LMR functioned as independent predictors for the occurrence of irAEs. (P=0.032; OR=0.246 [95% CI: 0.068-0.889] and P=0.020; OR=3.589 [95% CI: 1.223-10.537]). Patients exhibiting PLR ≤185 or LMR >4 had a higher likelihood of developing irAEs.

**Table 5:** Analysis of irAEs occurrence and influencing factors in 102 patients with stage IV driver gene-negative lung adenocarcinoma treated with ICIs.

Variable	No irAEs (N=65)	irAEs (N=37)	OR (95%CI)	P
CEA			0.859 (0.357-1.964)	0.718
≤ 5	24	15		
> 5	41	22		
CYFBA21-1			0.905 (0.372-2.204)	0.826
≤ 3.3	19	10		
>3.3	46	27		
NSE			1.252 (0.557-2.815)	0.587
≤ 16.3	38	18		
> 16.3	27	19		
SCC			1.494 (0.580-3.863)	0.404
≤ 1	46	29		
> 1	19	8		
D-dimer			0.482 (0.196-1.186)	0.112
≤ 0.5	39	28		
> 0.5	26	9		
NLR			0.199 (0.055-0.723)	0.014
≤ 5	45	34		
> 5	20	3		
PLR			0.125 (0.040-0.393)	<0.001
≤ 185	33	33		
> 185	32	4		
LMR			6.519 (2.615-16.249)	<0.001
≤ 4	44	9		
> 4	21	28		
PNI			1.115 (0.453-2.746)	0.813
≤ 45	19	10		
> 45	46	27		

## 4 Discussion

Although ICIs have demonstrated the capacity to enhance patient survival relative to conventional chemotherapy, a substantial proportion of lung adenocarcinoma patients remain unresponsive to these treatments. Consequently, it is imperative to identify the subset of lung adenocarcinoma patients who are more likely to derive benefit from immune checkpoint inhibitor (ICI) therapy.

The nutritional status and baseline immune function are critical determinants of treatment outcomes. Serum albumin serves as a straightforward yet valuable biomarker for assessing nutritional status, while lymphocyte counts provide insights into both chronic inflammatory conditions and immune functionality. The PNI, which integrates lymphocyte count with nutritional assessment, offers a more comprehensive reflection of the overall health condition of patients. Numerous studies have demonstrated that low PNI correlates with unfavorable prognoses in advanced NSCLC (18, 19). However, there is a paucity of research focusing on PNI in patients with stage IV driver gene-negative lung adenocarcinoma at diagnosis. This study aimed to explore the relationship between PNI and prognosis in such patients undergoing ICI therapy; results indicated that the 1-, 2-, and 3-year PFS rates and OS rates were significantly higher in the high PNI group compared to those in the low PNI group (P < 0.001, HR=0.407, 95%CI=0.234-0.708; P=0.001,HR=0.415, 95%CI=0.238-0.723. In recent years, the coagulation system has also been recognized as promoting the pro-tumor microenvironment of lung cancer (20, 21). A meta-analysis including seven studies with a total of 1,377 lung cancer patients demonstrated that those with low D-dimer levels had significantly longer survival times compared to their counterparts with high D-dimer levels (risk ratio for the high D-dimer group = 1.12; 95% confidence interval: 1.02-1.23). Overall survival was poorer in patients exhibiting elevated D-dimer levels relative to those with lower levels (22).

The concept of oligometastatic disease (OMD) was first proposed by Hellman and Weichselbaum to describe patients with a small number of metastatic lesions representing an intermediate state between localized and disseminated disease (23). The results of this study showed that the 1-, 2-, and 3-year PFS and OS rates in oligometastatic patients were 50%, 18.6%, and 5.7% and 85.7%, 34.3%, and 12.9%, respectively, which were significantly better than those in extensive metastatic patients, which were 3.1%, 0%, and 0% and 25%, 0%, and 0% (P < 0.001, HR=7.220, 95%CI=3.894-13.389; P < 0.001, HR=10.926, 95%CI=5.713-20.893), respectively. It is amply demonstrated that oligometastasis remains a significant prognostic factor for patients with stage IV driver gene-negative lung adenocarcinoma treated with ICIs. Populations with lower tumor loads may be an advantageous population to receive immunotherapy.

PD-1/PD-L1 inhibitors restore T-cell function and enhance the cytotoxic activity of T-cells against tumor cells by blocking the interaction between the PD-1 receptor on T-cells and its ligand, PD-L1 (24, 25). However, this mechanism may also lead to excessive activation of normal immune responses, resulting in an imbalance in immune system tolerance and irAEs (26). As the utilization of immune checkpoint inhibitors increases, so does the incidence of irAEs; severe cases can necessitate treatment interruption (27). Recent advancements in our understanding of irAEs have revealed a positive correlation between certain immune-related toxicities—such as skin reactions, hyperthyroidism, and hypothyroidism—and the efficacy of immunotherapies (28, 29). Notably, Owen DH et al. (30) reported that NSCLC patients who experienced irAEs while treated with nivolumab had improved OS compared to those who did not experience such events. Similarly, our analysis indicates that patients with irAEs exhibited longer PFS and OS, The 1-, 2- and 3-year PFS rates and OS rates of irAEs patients were 54.1%, 21.6%, 8.1% and 83.8%, 40.5%, and 21.6%, respectively, which were significantly better than 24.6%, 7.7%, 1.5% and 56.9%, 13.8%, and 1.5% without irAEs (P=0.002, HR=0.391, 95%CI=0.212-0.721; P < 0.001, HR=0.332, 95%CI=0.177-0.621). In this study cohort, the total incidence of irAEs was 36.3%, with immune-related rash (13.7%) and hypothyroidism (10.8%) being the most prevalent adverse effects. The occurrence rate for grade ≥ 3 irAEs was only 1.0%, which is generally lower than previously reported figures for similar groups. While rashes and hypothyroidism were predominant among these events, instances of immune-associated pneumonia were relatively rare at only 2.0%. This observation contributes to understanding why patients experiencing irAEs tend to have a more favorable prognosis.

This study also investigated the relationship between irAEs and peripheral hematological indicators as well as tumor markers in patients with stage IV driver gene-negative lung adenocarcinoma who were treated with ICIs. The findings revealed that a low PLR and a high LMR were significantly associated with the occurrence of irAEs, with P-values of 0.032 and 0.020, respectively. This suggests that monitoring the potential risk of irAEs in patients with low PLR and high LMR before treatment can help better manage irAEs, which is crucial for reducing the risk of hospitalization and treatment costs. Furthermore, it is also essential to explore the reasons why inflammatory factor indicators have predictive value regarding the occurrence of irAEs. Lymphocytes, particularly T cells, play a crucial role in anti-tumor immune responses and inhibit tumor cell proliferation. Following the administration of ICIs, lymphocytes become activated through interactions with self-antigens, which can result in damage to host tissues; this mechanism is primarily responsible for irAEs. A high LMR coupled with a low PLR indicates reduced peripheral blood monocyte and platelet counts alongside elevated lymphocyte levels. This profile suggests that the immune system is effectively sustaining anti-tumor responses while simultaneously heightening the risk of inappropriate lymphocytic reactions to self-antigens. Consequently, this may elucidate the increased incidence of irAEs observed in patients exhibiting higher LMR and lower PLR prior to treatment.

To our knowledge, this is the first survival and irAEs prediction to be developed for a population of stage IV driver gene-negative lung adenocarcinoma treated with ICIs. This model emphasizes the predictive significance of peripheral hematological indicators in relation to immune-related events. Furthermore, it offers a personalized, low-cost, and convenient approach to prediction. Despite these advantages, certain limitations must be acknowledged. As a single-center retrospective study, biases related to admission rates and information are unavoidable. Additionally, the incidence, types, and severity of irAEs may differ based on the specific immune checkpoint inhibitors utilized. Future research will continue to investigate survival outcomes and treatment-related adverse reactions among stage IV driver gene-negative lung adenocarcinoma patients receiving immunotherapy. Building upon this foundation, we will place greater emphasis on evaluating the efficacy of immunotherapy while thoroughly exploring clinical factors and biomarkers that predict its effectiveness—ultimately providing a more robust theoretical framework aimed at enhancing therapeutic efficacy while minimizing toxicities for these patients.

## 5 Conclusion

In summary, the PNI and metastatic status can serve as initial predictors of PFS in patients. Additionally, D-dimer levels, PNI, metastatic status, and the presence of irAEs can initially predict OS in stage IV driver gene-negative lung adenocarcinoma patients at the time of initial diagnosis who are treated with ICIs. Furthermore, the PLR and LMR may be valuable indicators for predicting irAEs in this patient population. Given the increasing focus on health-related costs and precision medicine, exploring the predictive role of peripheral blood markers in cancer immunotherapy is becoming increasingly significant. These preliminary findings warrant further investigation.

## Data Availability

The original contributions presented in the study are included in the article/Supplementary Material. Further inquiries can be directed to the corresponding author.
